# A novel compound heterozygous mutation in a Chinese boy with L-2-hydroxyglutaric aciduria: a case study

**DOI:** 10.1186/s12883-015-0369-2

**Published:** 2015-07-25

**Authors:** Hongfei Tai, Zaiqiang Zhang

**Affiliations:** Department of Neurology, Beijing Tiantan Hospital, Capital Medical University, 6 Tiantan Xili, dongcheng District, Beijing, China

**Keywords:** L-2-hydroxyglutaric aciduria, L-2-hydroxyglutarate dehydrogenase gene, Mutation, Phenotype

## Abstract

**Background:**

L-2-hydroxyglutaric aciduria is a rare autosomal recessive encephalopathy caused by mutations in the L-2-hydroxyglutarate dehydrogenase gene. We describe some novel clinical and molecular characteristics found in a boy with L-2-hydroxyglutaric aciduria.

**Case presentation:**

We report an 8-year-old Chinese boy, who had characteristic developmental delay, ataxia and acrocephaly as the main symptoms. He also complained of paroxysmal headache and palpitation. Brain image revealed a symmetrical, extensive subcortical white matter lesion. Urine test for organic acids showed a significantly increased level of 2-hydroxyglutaric acid (106.74 mmol/mol cre, normal range 0.6 ~ 5.9 mmol/mol cre), leading to the diagnosis of L-2-hydroxyglutaric aciduria. Genetic testing uncovered two heterozygous missense mutations in L-2-hydroxyglutarate dehydrogenase gene: c.169G > A in exon 2 and c.542G > T in exon 5, not hitherto been described.

**Conclusion:**

Novel gene mutation and associated clinical symptoms can contribute for the understanding and identification of this rare disease. Possible genotype-phenotype correlation waits for further study.

## Background

L-2-hydroxyglutaric aciduria is a rare autosomal recessive encephalopathy caused by mutations in the L-2-hydroxyglutarate dehydrogenase gene. Several clinical descriptions have appeared on L-2-hydroxyglutaric aciduria (L-2-HGA) since the index case reported in 1980 [1]. With insidious onset in childhood, the disease progresses slowly. The cardinal clinical signs include developmental delay, epilepsy and cerebellar ataxia, with half of the patients showing macrocephaly [2]. A mitochondrial enzyme, L-2-hydroxyglutarate dehydrogenase, catalyses the oxidation of L-2-hydroxyglutaric acid to alpha-ketoglutarate. Its functional defect leads to accumulation of L-2-hydroxyglutaric acid. Numerous mutations in L-2-hydroxyglutarate dehydrogenase gene have now been reported in L-2-HGA patients worldwide, registered in the Leiden Open Variation Database [3]. Currently, 92 unique variants have been described in 241 individuals who are homozygous or compound heterozygous for these alleles, and the majority of them are missense mutations. Here we describe some novel clinical and molecular characteristics found in a boy with L-2-hydroxyglutaric aciduria.

## Case presentation

An 8-year-old boy presented to our hospital for developmental delay and ataxia. He was born by cesarean section after fetal distress at 41 weeks gestation, with macrocephaly. He could not walk or speak a single word like ‘mom’ until 26 months of age. At approximately 4 years of age, his parents became concerned that he walked unstably, falling easily. He developed slight weakness and fatigue. In addition, he had paroxysmal headache and palpitation, aggravating muscle weakness. He had no convulsion, loss of consciousness, nausea, vomiting or blurred vision. During an episode of palpitation, lasting 2-3 minutes, the heart rate could reach 130-140 beats per minute, precipitating weakness, which resolved spontaneously in half an hour. His symptoms worsened continually. On entering a primary school at 8-years of age, he had difficulty keeping up with his peers. Family history reviewed no developmental delay, motor abnormalities or consanguinity. He had healthy parents and an older sister.

On examination, he was a little irritable. He showed normal orientation and comprehension for age, but could not do simple calculation. He had neither muscular hypertrophy nor atrophy, showing muscle strength of grade 5 minus (MRC), and normal muscle tone. He had symmetrical hyperreflexia and bilateral Babinski’s signs. He could not do well in tandem gait, or finger-to-nose and heel-knee-tibia test.

**Fig. 1:**
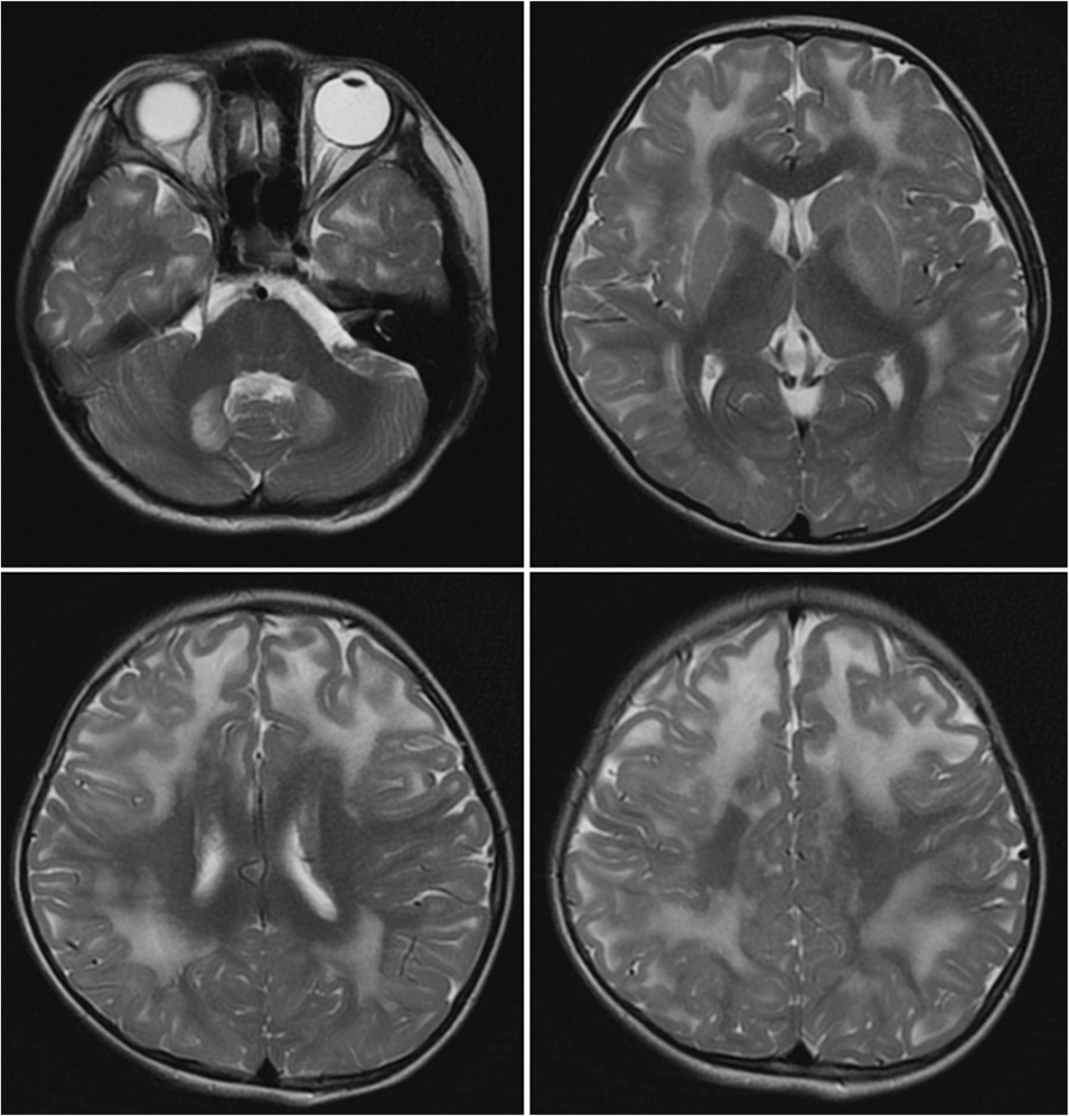
The patient’s brain magnetic resonance image (MRI). Axial T2-weighted sequence of the brain MRI showed symmetrical subcortical white matter hyperintense involving bilateral dentate nucleus, internal capsule, external capsule, and corona radiate

**Fig. 2:**
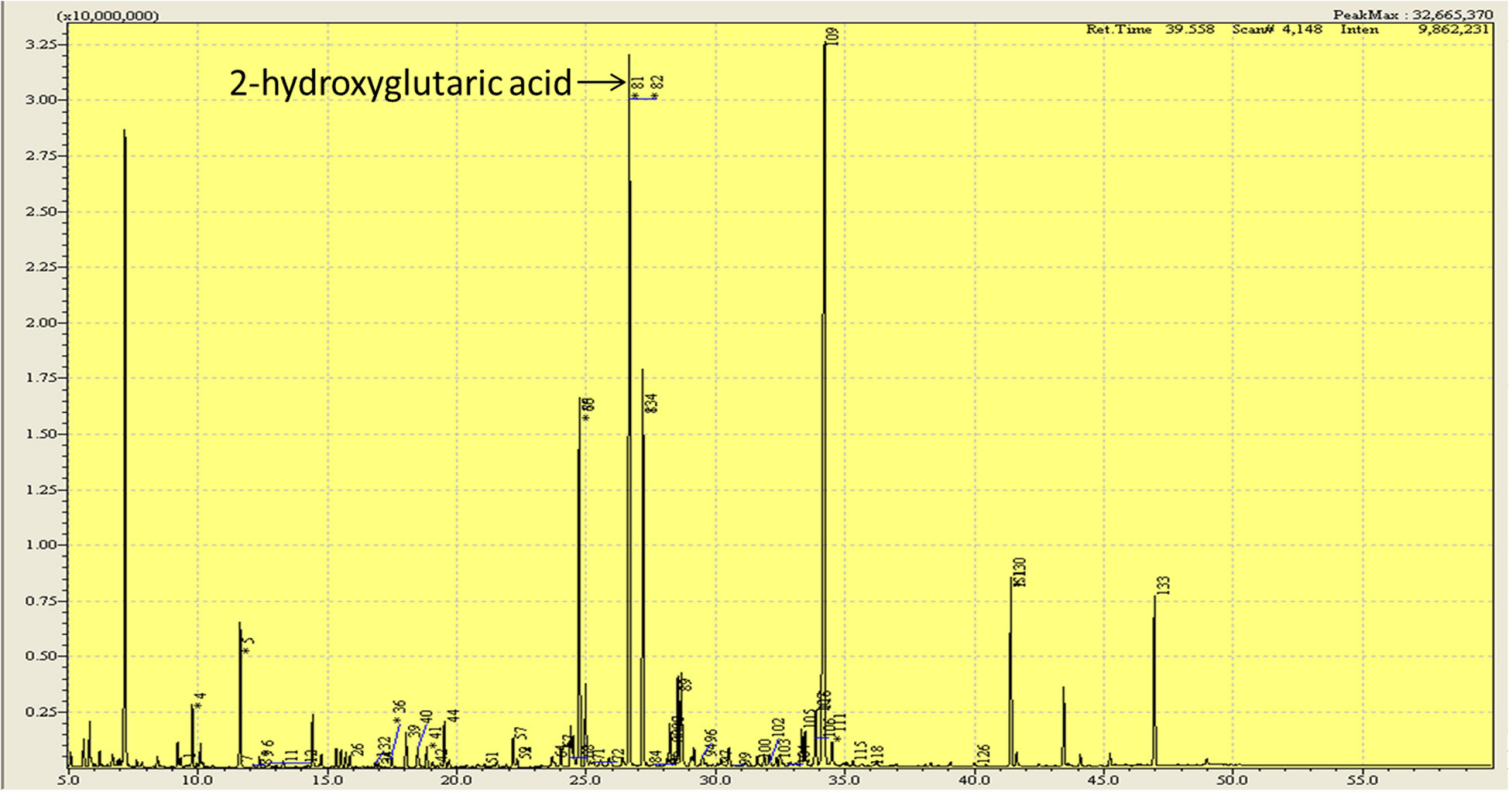
The patient’s urinary organic acid screening with GC-MS, showing increased 2-hydroxyglutaric acid concentrations

Brain MRI showed symmetrical, extensive subcortical hyperintense white matter lesion on T2-weighted sequences and diffusion-weighted imaging (DWI), involving bilateral dentate nucleus, internal capsule, external capsule and corona radiate (Fig. 1). Urinary organic acid screening with gas chromatography mass spectrometry (GC-MS) revealed significantly increased 2-hydroxyglutaric acid (106.74 mmol/mol cre, normal range 0.6 ~ 5.9 mmol/mol cre) (Fig. 2), 3-hydroxyglutaric acid (537.83 mmol/mol cre, nomal range 0) and 2- hexane acid (113.02 mmol/mol cre, normal range 0 ~ 16.4 mmol/mol cre), leading to the probable diagnosis of L-2-hydroxyglutaric aciduria.

**Fig. 3:**
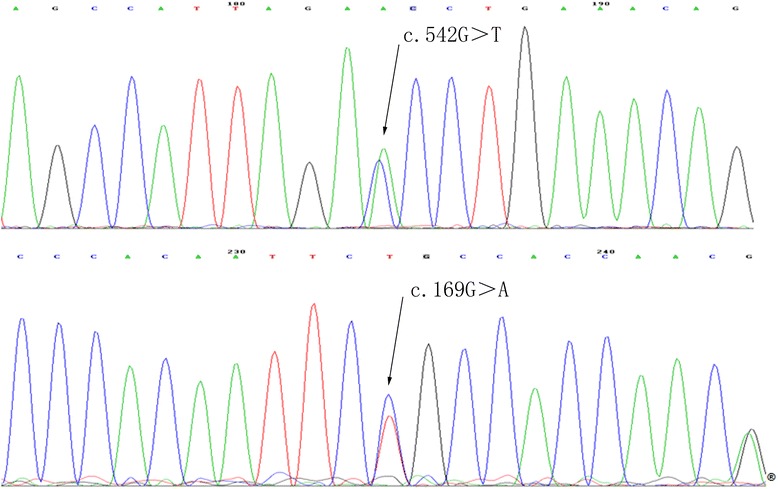
The patient’s L-2HGDH gene mutations. The patient’s L-2HGDH gene test showed two heterozygous mutations: c.542G > T in exon5 and c.169G > A in exon2

The patient received riboflavin 5 mg three times per day and oral L-carnitine 1 g per day, which stabilized the symptoms for one year. When he stopped the medication, the frequency of headache and palpitation increased. On his return 3 years later, brain MRI showed no obvious change. Genetic screening, conducted on this admission, identified two heterozygous mutations in the L-2-hydroxyglutarate dehydrogenase gene: G > T transversion (c.542G > T; p.G181V) in exon 5, and G > A transversion (c.169G > A; p.G57R) in exon 2 (Fig. 3). Familial gene analysis showed his father had the heterozygous mutation c.542G > T and his mother, the other heterozygous mutation c.169G > A.

## Conclusions

This patient had several characteristic symptoms such as developmental delay, ataxia, pyramidal impairment and macrocephaly, in combination with an elevated 2-hydroxyglutaric acid in urine. In gene test, he had a compound heterozygous missense mutation. The c.169G > A in exon 2 was firstly reported to be pathogenic in 2005 [4]. The other one c.542G > T in exon 5 was a novel mutation, which had not been reported before. The mutation located in conserved region. We used three tools including SIFT, PlyPhen and Mutationtaster to predict the possible impact of the amnio acid substitution on the structure and function of the protein L-2-hydroxyglutarate dehydrogenase. The test results indicated it was ‘deleterious (0 score), probably damaging (1 score) and disease causing’ respectively. This suggests that the mutation is probably to be pathogenic. Further functional test is in need.

Despite the general believe that the disease has highly homogeneous phenotype, our patient showed some novel symptoms. He had attacks of paroxysmal headache and palpitation, which worsened weakness. The accumulation of L-2-hydroxyglutaric acid causes leukoencephalopathy, with possible involvement of the autonomic nervous system, which may contribute to paroxysmal palpitation. Alternatively, paroxysmal palpitation and weakness may result from increased L-2-hydroxyglutaric acid accumulated in peripheral organs such as heart and muscle, in addition to the central nervous system as previous described [5] Possible genotype-phenotype correlation waits further investigation.

Supplementation with FAD and its precursor riboflavin may foster the residual enzymatic activity of L-2-hydroxyglutarate dehydrogenase with increased oxidation of L-2-hydroxyglutaric acid, thereby reducing its toxicity [6]. Previous studies showed a success with riboflavin (100 mg/day) and FAD (30 mg/day) therapy in some cases [7, 8], but not in others. In our patient, symptoms stabilized during the one year treatment, with its recurrence after discontinuation of therapy.

### Consent

Written informed consent was obtained from the patient for publication of this Case report and any accompanying images. A copy of the written consent is available for review by the Editor of this journal.

## Abbreviation

L-2HGA: L-2-hydroxyglutaric aciduria
